# Intramural cancer recurrence in the rectum after curative surgery for proximal sigmoid colon cancer: a case report

**DOI:** 10.1186/s12957-022-02794-w

**Published:** 2022-10-03

**Authors:** Yusuke Asada, Katsuya Chinen, Ken Yamataka, Jo Tokuyama, Naoto Kurihara, Shuhei Iida

**Affiliations:** 1 Department of Surgery, Nerima General Hospital, 1-24-1 Asahigaoka, Nerima, Tokyo, 176-8530 Japan; 2 Department of Pathology, Nerima General Hospital, 1-24-1 Asahigaoka, Nerima, Tokyo, 176-8530 Japan

**Keywords:** Intramural metastasis, Colon cancer, Distal intramural spread, Tumor-specific mesorectal excision, Local recurrence

## Abstract

**Background:**

Intramural metastasis distant from the primary tumor is rare in colorectal cancer. Here, we present a notably rare and probably the first case of asynchronous intramural recurrence in the rectum after curative surgery for proximal sigmoid colon cancer.

**Case presentation:**

A 44-year-old man underwent curative sigmoidectomy for proximal sigmoid colon cancer with T3N0M0, Stage IIA tubular adenocarcinomas. After 15 months, the tumor marker level had increased, and positron emission tomography-computed tomography (PET-CT) revealed abnormal fluorodeoxyglucose uptake in the rectum; colonoscopy revealed a submucosal tumor (SMT)-like lesion in the upper rectum, and biopsy revealed a tubular adenocarcinoma. We performed curative low anterior resection with tumor-specific mesorectal excision (TSME). The SMT-like tumor was located approximately 20 cm from the initial sigmoid colon anastomosis (i.e., at least 20 cm distal to the initial sigmoid colon cancer). The pathological findings revealed cancer cells with the same features as the initial sigmoid colon cancer, only in the intestinal wall but not in the mucosa and extramural tissue. Therefore, the lesion was determined to be an intramural recurrence.

After 24 months, lung recurrence, and local recurrence, which might have involved the lymph nodes in the preserved mesorectum after TSME at the bottom of the pelvis was detected on PET-CT. Hence, we started systemic chemotherapy.

**Conclusions:**

This case report suggests that PET-CT and short-interval repeat colonoscopy may help detect a rare intramural recurrence. A long distal margin may be necessary to achieve local control in the rectal resection for intramural recurrence.

## Background

Intramural metastasis distant from the primary tumor is rare in colorectal cancer. Here, we present a notably rare case of asynchronous intramural recurrence in the rectum after curative surgery for proximal sigmoid colon cancer. To the best of our knowledge, this paper is the first case report of this type of recurrence.

## Case presentation

**Fig. 1:**
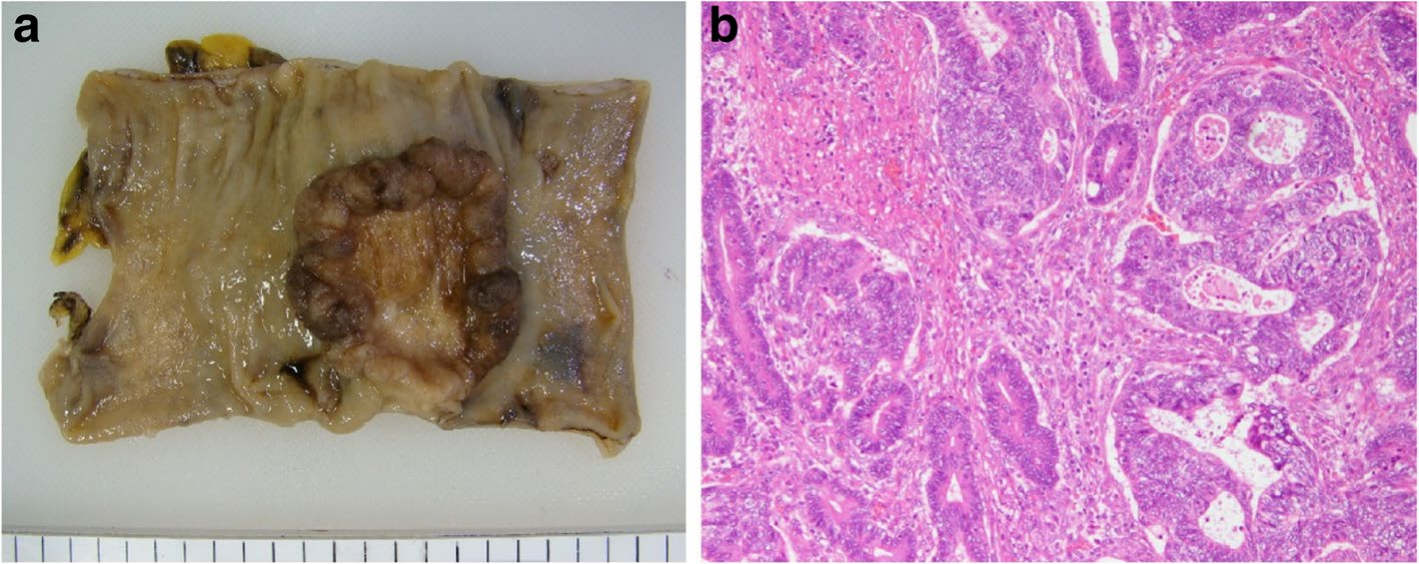
Specimen and pathological findings in the initial surgery. Curative sigmoidectomy for proximal sigmoid colon cancer was performed (**a**), and pathological findings revealed tubular adenocarcinoma (HE, ×10) (**b**)

A 44-year-old man with no specific medical or family history underwent curative sigmoidectomy for proximal sigmoid colon cancer with tubular adenocarcinoma. We performed standard regional lymph node dissection with high ligation of the inferior mesenteric artery and functional end-to-end anastomosis. The pathological diagnosis was T3N0M0, Stage IIA, according to the 8^th^ Union for International Cancer Control classification. Moderate venous invasion was observed; however, lymphatic invasion was unnoted. Sufficient proximal and distal margins from the tumor were maintained (Fig. 1). Carcinoembryonic antigen (CEA) levels became normal after the surgery (from 11.0 to 3.3 ng/mL). No adjuvant chemotherapy was administered.

**Fig. 2:**
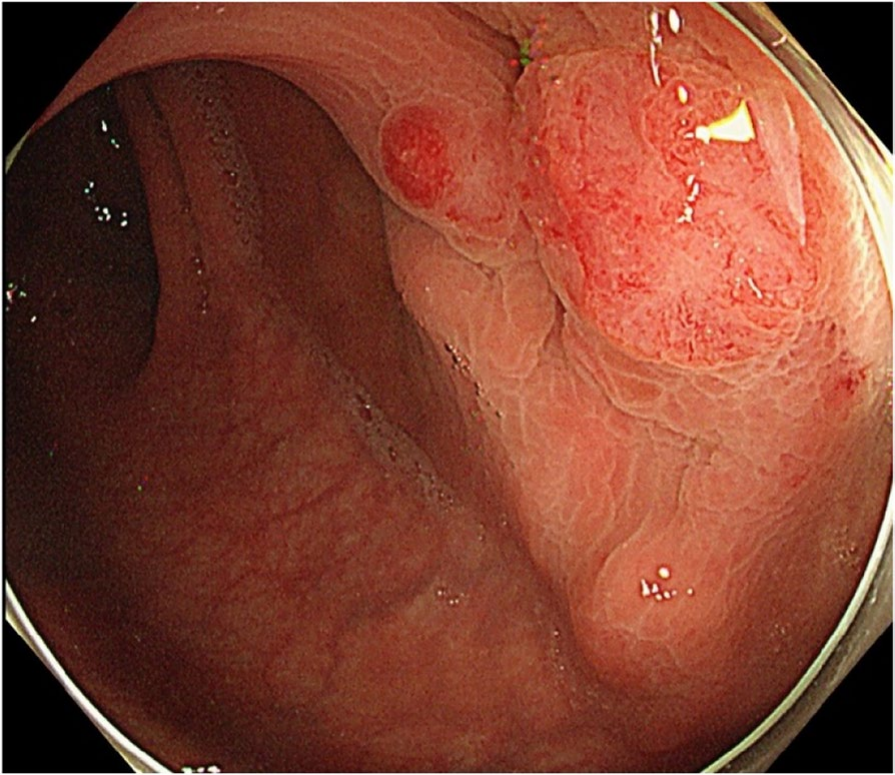
Colonoscopy findings of intramural recurrence. An SMT-like lesion was observed in the upper rectum, and biopsy revealed tubular adenocarcinoma

**Fig. 3:**
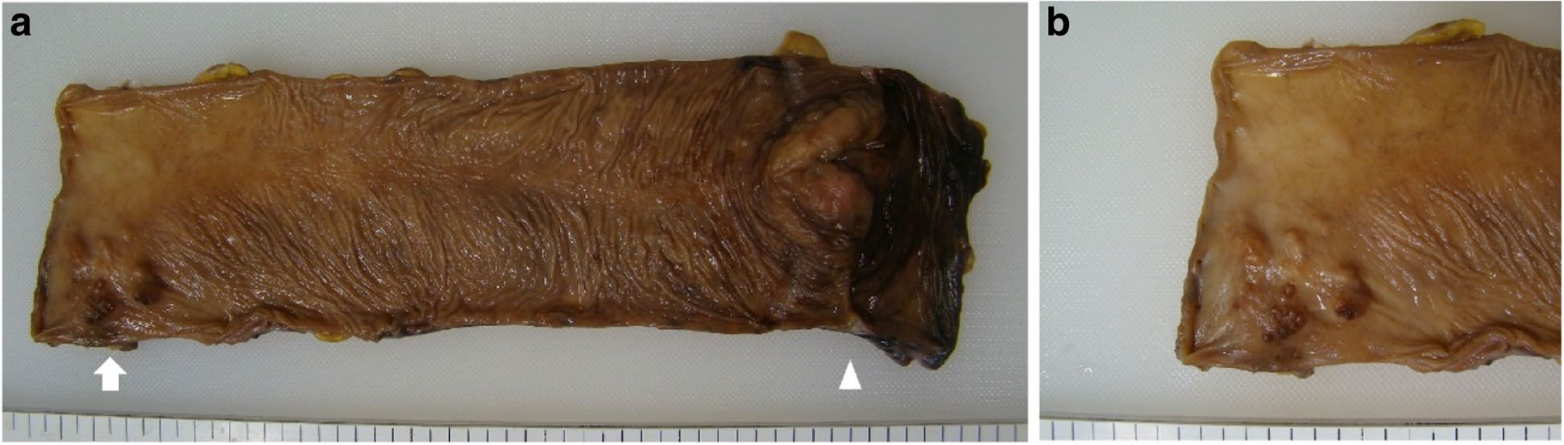
Specimen from the secondary surgery (LAR for intramural recurrence). A recurrent lesion (arrow) was located approximately 20 cm from the initial sigmoid colon anastomosis (arrowhead) (i.e., at least 20 cm distal to the initial sigmoid colon cancer) (**a**). The lesion resembled an SMT (**b**)

**Fig. 4:**
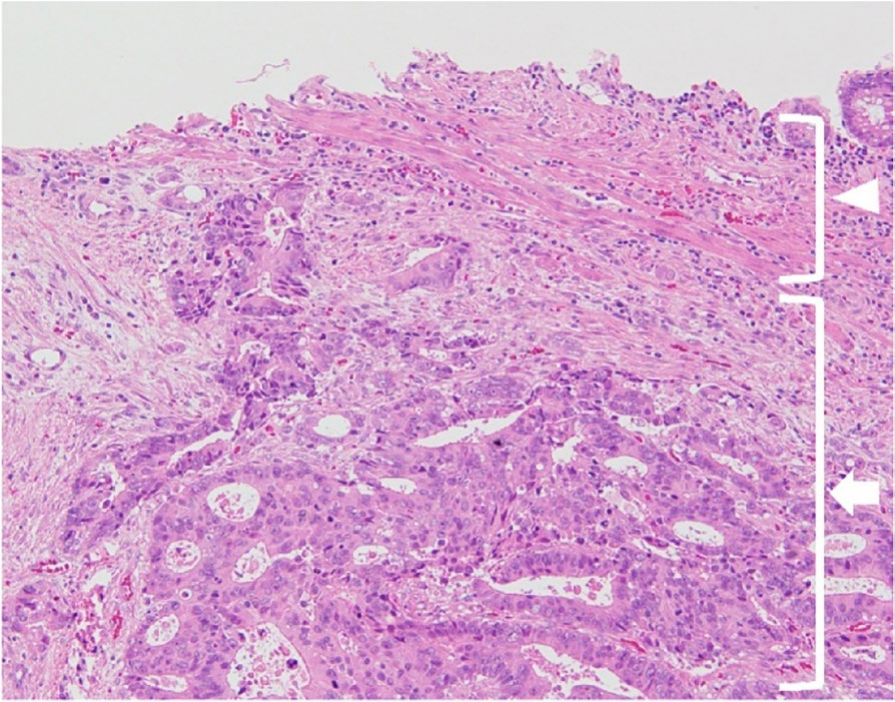
Pathological findings of intramural recurrence. Cancer cells with the same feature as the initial sigmoid colon cancer were observed only in the intestinal wall (arrow), but not in the mucosa (arrowhead) (HE, ×10)

After 12 months, the CEA level increased to 9.0 ng/mL, although no recurrence was detected on computed tomography (CT) and colonoscopy. After another 3 months, the CEA level further increased to 17.2 ng/mL. We performed positron emission tomography-CT (PET-CT), and abnormal fluorodeoxyglucose uptake was detected in the rectum. Repeat colonoscopy revealed a submucosal tumor (SMT)-like lesion in the upper rectum (10–15 cm from the anal verge), and biopsy revealed tubular adenocarcinoma (Fig. 2). Lymph node and distant metastases were not identified on CT and PET-CT. We performed curative low anterior resection (LAR) with double-stapled anastomosis under the differential diagnosis of an atypical primary cancer or a rare case of intramural recurrence. We chose tumor-specific mesorectal excision (TSME) with a distal margin of 3 cm from the tumor. The postoperative course was uneventful. The SMT-like tumor was located approximately 20 cm from the initial sigmoid colon anastomosis in the specimen (i.e., at least 20 cm distal from the initial sigmoid colon cancer) (Fig. 3). Pathological findings revealed cancer cells with the same features as the initial sigmoid colon cancer, only in the intestinal wall but not in the mucosa and extramural tissue (Fig. 4). These findings were unlikely of primary cancer or lymph node recurrence, and likely of an intramural recurrence. Therefore, we determined that the lesion was an intramural recurrence from the initial sigmoid colon cancer. Severe venous invasion was also observed; however, lymphatic invasion was not observed. All resection margins were negative, and the CEA level became normal again (4.3 ng/mL). The RAS and BRAF statuses were both wild-type.

**Fig. 5:**
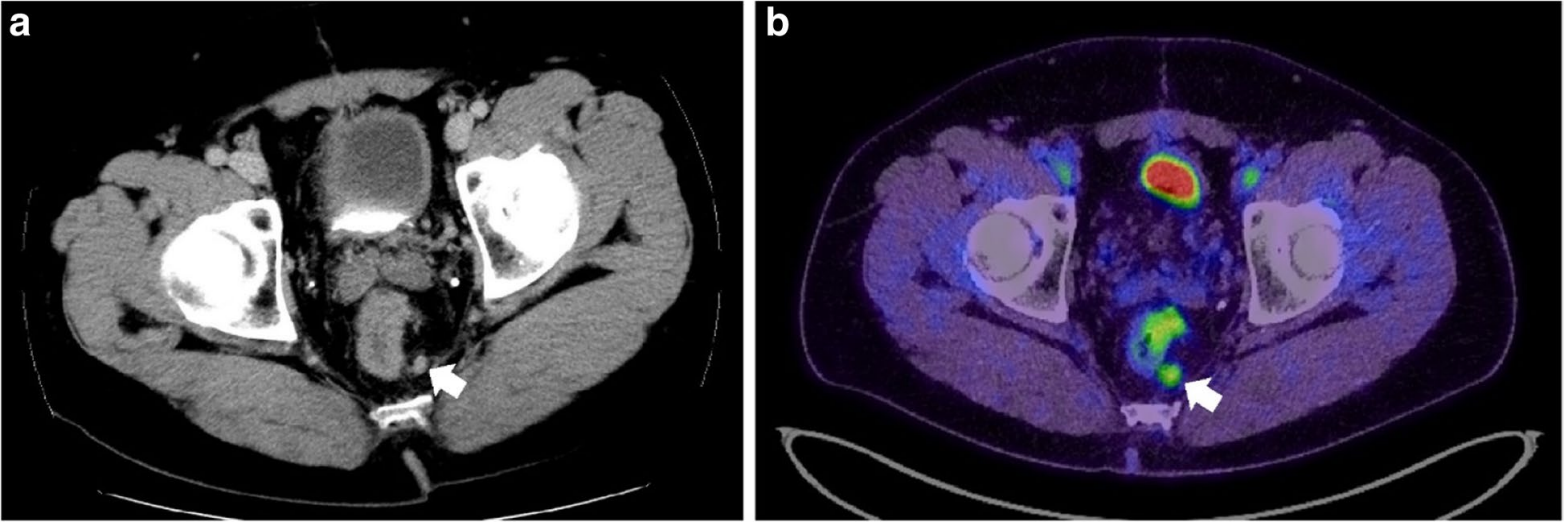
Imaging of local recurrence after secondary LAR for intramural recurrence. CT detected a small nodule which may have been lymph node recurrence in the preserved mesorectum after TSME at the bottom of the pelvis (arrow) (**a**). Abnormal fluorodeoxyglucose uptake was observed on PET-CT (arrow) (**b**)

We planned adjuvant chemotherapy with eight courses of capecitabine plus oxaliplatin (CapeOX). However, after three courses, the CEA levels increased again, and a single lung recurrence was detected; this lung lesion was resected and CapeOX therapy was not used. Twelve months after pneumonectomy, another lung recurrence and a local recurrence, which may have been related to recurrence in the lymph node in the preserved mesorectum after TSME at the bottom of the pelvis, were detected on CT and PET-CT (Fig. 5). We initiated FOLFIRI (5-fluorouracil, l-leucovorin, and irinotecan) plus panitumumab for unresectable recurrent colorectal cancer. Stable disease was maintained after 14 courses (51 months from the initial sigmoidectomy and 36 months from the secondary LAR).

## Discussion and conclusions

In esophageal cancer, intramural metastasis is a well-known metastatic lesion in the gastrointestinal wall and is clearly distant from the primary tumor [1]. A similar etiology in colorectal cancer is known as distal intramural spread (DIS), which is sometimes an unexpected cancer in the distal intestinal wall under the normal mucosa; DIS comprises both continuous spread from the primary tumor and skip lesions [2–4]. DIS itself is not rare and is observed in approximately 25% of resected rectal cancer specimens, but the maximum DIS distance from the primary tumor is within 2 cm in almost all cases [2, 5]. Only a few case reports of distant DIS (approximately 6 cm) have been published [3, 4]. Furthermore, DIS is usually related to rectal cancer, not colon cancer, and there is only one case report regarding intramural cancer spread in colon cancer [6]. This report presented a rare case of asynchronous intramural recurrence in the rectum following curative surgery for proximal sigmoid colon cancer. Recurrence occurred from a very distant DIS, at least 20 cm from the primary tumor.

This type of recurrence due to distant DIS occurs from vessel (lymphatic and venous) invasion, although cancer cell implantation from an inadequate surgical procedure may result in intramural recurrence near the primary surgical site [2, 3, 7]. Moderate to severe venous invasion was observed in both the primary and recurrent tumors in our case. The venous pathway for long DIS is consistent with a high rate of hematogenous recurrence in the liver and lung after curative surgery for distant DIS cases in previous reports and lung recurrence in our case [3, 5].

The diagnostic flow of intramural recurrence in our case is important. The initial colonoscopy did not reveal any abnormal findings, but a repeat colonoscopy after only 3 months showed recurrence. Intramural cancer lesions, which usually grow as SMT-like lesions, are difficult to identify on colonoscopy as long as they result in mucosal changes. In fact, there are only a few case reports of the intramural spread of colorectal cancer that could be diagnosed via colonoscopy [7, 8]. Furthermore, significant changes in intramural lesions at short intervals, such as in our case, were also observed in a previous report, which reported the appearance of intramural lesions only 5 weeks after negative findings on initial colonoscopy [3]. To detect intramural recurrence without delay, other modalities such as PET-CT and short-interval repeat colonoscopy may be considered if there are compatible reasons (e.g., the inexplicable elevation of tumor markers such as in our case).

The appropriateness of our surgical procedure for intramural recurrence in the rectum, LAR with TSME, is another concern. Although a favorable sphincter-preserving surgery without postoperative complications was achieved, undesirable local recurrence, which might have involved the lymph nodes in the preserved mesorectum after TSME, was observed. In fact, distal cancer spread is observed not only intramurally (i.e., DIS), but also extramurally (i.e., mesorectum); thus, adequate resection of the distal mesorectum is key to avoiding local recurrence in rectal cancer surgery [9, 10]. Total mesorectal excision (TME), which involves the resection of the entire mesorectum and may sometimes contain unexpected cancer spread, is the conventional gold-standard procedure for rectal cancer [11, 12]. However, a high anastomotic leakage rate and unnecessary sphincter loss are serious concerns in TME; therefore, TSME with a 2–3-cm distal margin from the tumor is widely accepted, especially in upper rectal cancer [13]. Several studies have reported that distal cancer spread (both intramural and extramural) was within 2–3 cm from the primary tumor in almost all cases, indicating the appropriateness of TSME [9, 10, 14]. However, such a consensus is currently for normal primary cancer, and intramural recurrence, such as in our case, is expected to have a longer distal cancer spread than in usual cases. Although it is difficult to make a definitive diagnosis of intramural recurrence before surgery, TME may be the preferred procedure to achieve local control if intramural recurrence is noted from preoperative findings, even if abdominoperineal resection with permanent colostomy is required.

In conclusion, we present a rare case of asynchronous intramural recurrence in the rectum after curative surgery for proximal sigmoid colon cancer. We suggest the following two take-home messages from this report. First, PET-CT and short-interval repeat colonoscopy may be useful to detect intramural recurrence. Second, a longer distal margin than usual may be necessary to achieve local control in the rectal resection for intramural recurrence.

## Data Availability

Not applicable.

## Abbreviations

CEA: Carcinoembryonic antigen
CT: Computed tomography
PET-CT: Positron emission tomography-computed tomography
SMT: Submucosal tumor
LAR: Low anterior resection
TSME: Tumor-specific mesorectal excision
DIS: Distal intramural spread
TME: Total mesorectal excision
